# A methylome-wide association study of major depression with out-of-sample case–control classification and trans-ancestry comparison

**DOI:** 10.1038/s44220-025-00486-4

**Published:** 2025-09-16

**Authors:** Xueyi Shen, Miruna Barbu, Doretta Caramaschi, Ryan Arathimos, Darina Czamara, Friederike S. David, Anna Dearman, Evelyn Dilkes, Marisol Herrera-Rivero, Floris Huider, Luise Kühn, Kuan-Chen Lu, Teemu Palviainen, Alicia M. Schowe, Gemma Shireby, Antoine Weihs, Chloe C. Y. Wong, Eleanor Davyson, Hannah Casey, Mark J. Adams, Antje-Kathrin Allgaier, Michael Barber, Joe Burrage, Avshalom Caspi, Ricardo Costeira, Erin C. Dunn, Lisa Feldmann, Josef Frank, Franz J. Freisleder, Danni A. Gadd, Ellen Greimel, Eilis Hannon, Sarah E. Harris, Georg Homuth, David M. Howard, Stella Iurato, Tellervo Korhonen, Tzu-Pin Lu, Nicholas G. Martin, Jade Martins, Edel McDermott, Susanne Meinert, Pau Navarro, Miina Ollikainen, Verena Pehl, Charlotte Piechaczek, Aline D. Scherff, Frederike Stein, Fabian Streit, Alexander Teumer, Henry Völzke, Jenny van Dongen, Rosie M. Walker, Natan Yusupov, Louise Arseneault, Jordana T. Bell, Klaus Berger, Elisabeth Binder, Dorret I. Boomsma, Simon R. Cox, Udo Dannlowski, Kathryn L. Evans, Helen L. Fisher, Andreas J. Forstner, Hans J. Grabe, Jaakko Kaprio, Tilo Kircher, Johannes Kopf-Beck, Meena Kumari, Po-Hsiu Kuo, Qingqin S. Li, Terrie E. Moffitt, Hugh Mulcahy, Therese M. Murphy, Gerd Schulte-Körne, Jonathan Mill, Cathryn M. Lewis, PGC MDD Working Group, Xueyi Shen, Doretta Caramaschi, Ryan Arathimos, Darina Czamara, Friederike S. David, Anna Dearman, Evelyn Dilkes, Marisol Herrera-Rivero, Floris Huider, Luise Kühn, Kuan-Chen Lu, Teemu Palviainen, Alicia M. Schowe, Gemma Shireby, Antoine Weihs, Chloe C. Y. Wong, Mark J. Adams, Antje-Kathrin Allgaier, Michael Barber, Joe Burrage, Avshalom Caspi, Ricardo Costeira, Erin C. Dunn, Lisa Feldmann, Josef Frank, Franz J. Freisleder, Ellen Greimel, Eilis Hannon, Georg Homuth, David M. Howard, Stella Iurato, Tellervo Korhonen, Tzu-Pin Lu, Nicholas G. Martin, Jade Martins, Edel McDermott, Susanne Meinert, Miina Ollikainen, Verena Pehl, Charlotte Piechaczek, Aline D. Scherff, Frederike Stein, Fabian Streit, Alexander Teumer, Henry Völzke, Natan Yusupov, Louise Arseneault, Jordana T. Bell, Klaus Berger, Elisabeth Binder, Dorret I. Boomsma, Udo Dannlowski, Helen L. Fisher, Andreas J. Forstner, Hans J. Grabe, Jaakko Kaprio, Tilo Kircher, Johannes Kopf-Beck, Meena Kumari, Po-Hsiu Kuo, Qingqin S. Li, Terrie E. Moffitt, Hugh Mulcahy, Therese M. Murphy, Gerd Schulte-Körne, Cathryn M. Lewis, Jenny van Dongen, Naomi R. Wray, Andrew M. McIntosh, Naomi R. Wray, Andrew M. McIntosh

**Affiliations:** 1 Institute of Neuroscience and Cardiovascular Research, University of Edinburgh, Edinburgh, UK; 2 Faculty of Health and Life Sciences, Department of Psychology, University of Exeter, Exeter, UK; 3 Social, Genetic and Developmental Psychiatry Centre, Institute of Psychiatry, Psychology and Neuroscience, King’s College London, London, UK; 4 Department Genes and Environment, Max-Planck-Institute of Psychiatry, Munich, Germany; 5 Institute of Human Genetics, University of Bonn, School of Medicine and University Hospital Bonn, Bonn, Germany; 6 Institute for Social and Economic Research, University of Essex, Colchester, UK; 7 Department of Genetic Epidemiology, Institute of Human Genetics, University of Münster, Münster, Germany; 8 Institute of Epidemiology and Social Medicine, University of Münster, Münster, Germany; 9 Biological Psychology, Amsterdam Public Health Institute, Vrije Universiteit Amsterdam, Amsterdam, the Netherlands; 10 Department of Psychiatry and Psychotherapy, University Medicine Greifswald, Greifswald, Germany; 11 Institute of Epidemiology and Preventive Medicine, National Taiwan University, Taipei, Taiwan; 12 Institute for Molecular Medicine Finland—FIMM, University of Helsinki, Helsinki, Finland; 13 Ludwig-Maximilians-Universität, Graduate School of Systemic Neuroscience, Munich, Germany; 14 University of Exeter Medical School, University of Exeter, Exeter, UK; 15 Institute of Education, University College London, London, UK; 16 German Centre for Neurodegenerative Diseases (DZNE), Greifswald, Germany; 17 School of Informatics, University of Edinburgh, Edinburgh, UK; 18 Department of Human Sciences, University of the Bundeswehr Munich, Munich, Germany; 19 MRC Human Genetics Unit, University of Edinburgh, Edinburgh, UK; 20 Department of Clinical and Biomedical Sciences, University of Exeter, Exeter, UK; 21 Departments of Psychology and Neuroscience, Psychiatry and Behavioral Sciences, and Institute for Genome Sciences and Policy, Duke University, Durham, NC USA; 22 Department of Twin Research and Genetic Epidemiology, King’s College London, London, UK; 23 Psychiatric and Neurodevelopmental Genetics Unit, Center for Genomic Medicine, Massachusetts General Hospital, Boston, MA USA; 24 Department of Psychiatry, Harvard Medical School, Boston, MA USA; 25 Department of Child and Adolescent Psychiatry, Psychosomatics and Psychotherapy, LMU University Hospital, Ludwig-Maximilians-University (LMU) Munich, Munich, Germany; 26 German Center for Mental Health (DZPG), Partner Site Munich-Augsburg, Munich, Germany; 27 Department of Genetic Epidemiology in Psychiatry, Central Institute of Mental Health, Medical Faculty Mannheim, Heidelberg University, Mannheim, Germany; 28 kbo-Heckscher-Klinikum gGmbH, Munich, Germany; 29 Institute of Genetics and Cancer, Centre for Genomic and Experimental Medicine, University of Edinburgh, Edinburgh, UK; 30 Lothian Birth Cohorts group, Department of Psychology, University of Edinburgh, Edinburgh, UK; 31 Interfaculty Institute for Genetics and Functional Genomics, University Medicine Greifswald, Greifswald, Germany; 32 Roche Diagnostics GmbH, Munich, Germany; 33 Department of Public Health and Institute of Health Data Analytics and Statistics, National Taiwan University, Taipei, Taiwan; 34 Queensland Institute of Medical Research Berghofer, Brisbane, Queensland Australia; 35 Decision Intelligence, Knorr-Bremse Services GmbH, Munich, Germany; 36 Centre for Colorectal Disease, St Vincent’s University Hospital, Dublin, Ireland; 37 Institute for Translational Psychiatry, University of Münster, Münster, Germany; 38 Institute for Translational Neuroscience, University of Münster, Münster, Germany; 39 The Roslin Institute, University of Edinburgh, Edinburgh, UK; 40 Minerva Foundation Institute for Medical Research, Helsinki, Finland; 41 Department of Psychiatry and Psychotherapy, University of Marburg, Marburg, Germany; 42 Center for Mind, Brain and Behavior (CMBB), University of Marburg, Marburg, Germany; 43 Hector Institute for Artificial Intelligence in Psychiatry, Central Institute of Mental Health, Medical Faculty Mannheim, Heidelberg University, Mannheim, Germany; 44 Department of Psychiatry and Psychotherapy, Central Institute of Mental Health, Medical Faculty Mannheim, Heidelberg University, Mannheim, Germany; 45 German Center for Mental Health (DZPG), Partner Site Mannheim-Heidelberg-Ulm, Mannheim, Germany; 46 German Centre for Cardiovascular Research (DZHK), Greifswald, Germany; 47 Institute for Community Medicine, University Medicine Greifswald, Greifswald, Germany; 48 International Max Planck Research School for Translational Psychiatry (IMPRS-TP), Munich, Germany; 49 Complex Trait Genetics, Center for Neurogenomics and Cognitive Research, Vrije Universiteit Amsterdam, Amsterdam, the Netherlands; 50 Institute for Genetics and Cancer, University of Edinburgh, Edinburgh, UK; 51 ESRC Centre for Society and Mental Health, King’s College London, London, UK; 52 Institute of Neuroscience and Medicine (INM-1), Research Center Jülich, Jülich, Germany; 53 Centre for Human Genetics, University of Marburg, Marburg, Germany; 54 Clinical Department, Max Planck Institute of Psychiatry, Munich, Germany; 55 Department of Psychology, LMU Munich, Munich, Germany; 56 Department of Public Health & Institute of Epidemiology and Preventive Medicine, National Taiwan University, Taipei, Taiwan; 57 Department of Psychiatry, National Taiwan University Hospital, Taipei, Taiwan; 58 Neuroscience, Janssen Research and Development, Titusville, NJ USA; 59 School of Medicine, University College Dublin, Dublin, Ireland; 60 School of Biological, Health and Sports Sciences, Technological University Dublin, Dublin, Ireland; 61 Institute for Molecular Bioscience, University of Queensland, Brisbane, Queensland Australia; 62 Department of Psychiatry, University of Oxford, Oxford, UK

**Keywords:** Predictive markers, DNA methylation, Depression, Epigenomics

## Abstract

Major depression (MD) is a leading cause of global disease burden, and both experimental and population-based studies suggest that differences in DNA methylation may be associated with the condition. However, previous DNA methylation studies have, so far, not been widely replicated, suggesting a need for larger meta-analysis studies. Here we conducted a meta-analysis of methylome-wide association analysis for lifetime MD across 18 studies of 24,754 European-ancestry participants (5,443 MD cases) and an East Asian sample (243 cases, 1,846 controls). We identified 15 CpG sites associated with lifetime MD with methylome-wide significance. The methylation score created using the methylome-wide association analysis summary statistics was significantly associated with MD status in an out-of-sample classification analysis (area under the curve 0.53). Methylation score was also associated with five inflammatory markers, with the strongest association found with tumor necrosis factor beta. Mendelian randomization analysis revealed 23 CpG sites potentially causally linked to MD, with 7 replicated in an independent dataset. Our study provides evidence that variations in DNA methylation are associated with MD, and further evidence supporting involvement of the immune system.

## Abstract (web summary)

The authors report a meta-analysis of methylome-wide association studies, identifying 15 significant CpG sites linked to major depression, revealing associations with inflammatory markers and suggesting potential causal relationships through Mendelian randomization analysis.

## Main

Major depression (MD) is a common psychiatric disorder arising from a complex combination of genetic and environmental factors that include lifestyle factors such as physical activity, smoking, alcohol consumption and body mass index (BMI)^1–3^. The heritability of MD estimated from twin studies is 37% (ref. ^4^), and polygenic risk scores trained on genome-wide association studies (GWASs) currently explain 1.5–3.2% of the variance in MD^3^.

DNA methylation (DNAm) is one of the most studied epigenetic processes and is influenced by both genetic and environmental factors^5^. DNAm is dynamic and is associated with changes in environmental and lifestyle factors, including smoking^6^, alcohol^7^ and BMI^8^, all factors that are implicated in MD^3^. A recent study^9^ identified associations between methylation scores (MSs), calculated using methylome-wide association study (MWAS) summary statistics for several relevant lifestyle factors, and MD^10^. These environmental MS measures were able to capture additional variation associated with MD when added to direct lifestyle measures, and it is thought this may be due to their ability to act as an archive of environmental exposure.

There is growing evidence from MWAS that DNAm measured in whole blood may be associated with MD. Jovanova et al.^11^ looked at 11 cohorts comprising 11,256 participants of European and African ancestry and identified 3 cytosine–phosphate–guanine (CpG) sites associated with depressive symptoms, which were annotated to genes implicated in axon guidance^11^. Starnawska et al.^12^ investigated depressive symptomatology in a sample of 724 monozygotic twins, with top findings annotated to genes previously implicated in depression^12^. Finally, Huls et al.^13^ identified DNAm associations with MD in dorsal lateral prefrontal cortex samples from *N* = 608 participants and uncovered CpGs annotated to several genes that are relevant to MD^13^. In addition, MS calculated using penalized regression models have previously been utilized to investigate MD^14^. We recently used lasso regression to calculate an MD–MS in *N* = 1,780 participants (cases = 363; controls = 1,417) and found that the scores explain approximately 1.75% of the variance in prevalent MD, acting additively with polygenic risk score^14^. Findings from these studies have been somewhat inconsistent, and they have either included only European-ancestry samples^15,16^ or used a combined multi-ancestry sample without considering trans-ancestry differences^11^. This has limited generalizability of previous findings. Larger MWAS studies may provide more reliable estimates of the differences in DNAm between MD cases and controls and, in doing so, bring insight into the molecular mechanisms associated with MD. We further hypothesized that DNAm associations with MDD would generalize across ancestries and sought to test this in a case–control sample of East Asian ancestry.

Given previous evidence highlighting the role of DNAm in MD, we conducted an MD MWAS meta-analysis using whole-blood DNAm data from 18 cohorts comprising 24,754 individuals (5,443 cases) of European ancestry. We sought to identify whether differences in DNAm were a potential cause or consequence of MD using a two-sample Mendelian randomization framework. Further, we trained a DNAm classifier of MD status from our summary statistics and assessed whether it could classify MD case–control status in an independent testing sample and its association with the abundance of inflammatory protein markers. Finally, we assessed whether significant effect sizes were positively associated with those in an independent East Asian sample.

## Results

### MD meta-MWAS

**Fig. 1:**
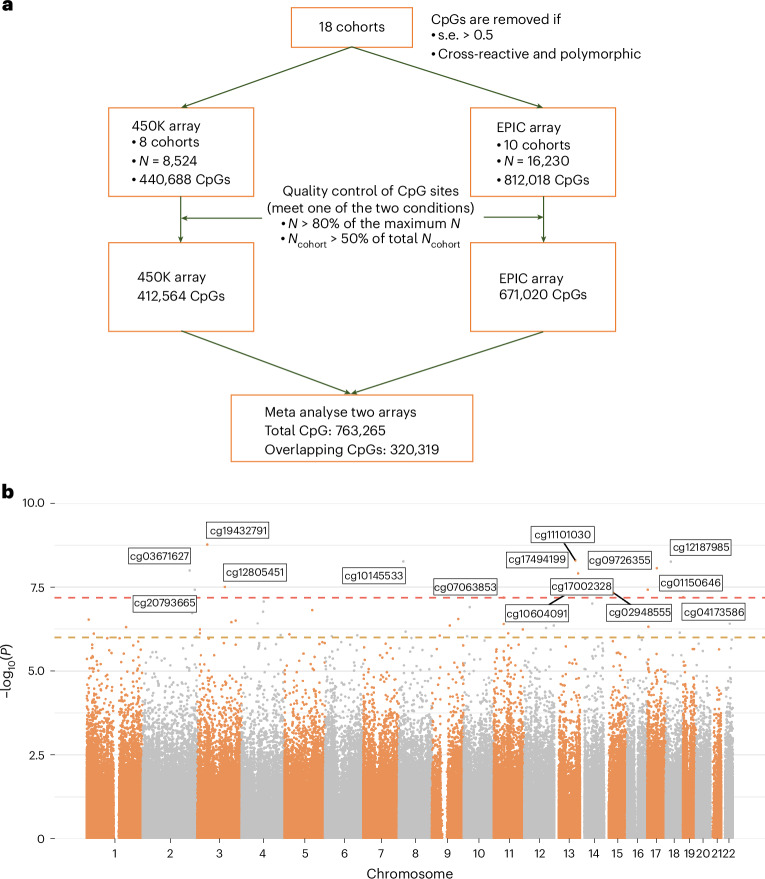
Meta-analysis of MWAS for MD. **a**, Workflow of meta-analysis. **b**, Manhattan plot for the meta-analysis of MWAS for MD. Each dot represents a CpG probe. The *x* axis represents the relative positions of the probes in the genome. The *y* axis represents −log_10_-transformed, two-sided *P* values. The red and yellow dashed lines represent the significance threshold for Bonferroni and FDR correction, respectively.

A total of 15 CpG sites were significantly associated with MD after Bonferroni correction in the basic model (*P* < 6.55 × 10^−8^, *P*_Bonferroni_ < 0.05; see Table 1 for details of participating studies and Fig. 1 and Table 2 for significant findings).

**Table 1:** Information for cohorts that participated in the methylome-wide meta-analysis

Cohort name	PMID	*N* cases	Sample size	MD prevalence (%)	Case phenotype	Mean age	Gender (% male)	Genetic ethnicity	DNAm array	Tissue type
Avon Longitudinal Study of Parents and Children/Accessible Resource for Integrated Epigenomic Studies	25991711	204	657	31.1	Questionnaire	29.4/45	0	European	450K	Whole blood
The biological classification of mental disorders/the Optimized Treatment Identification at the Max Planck Institute	32393358; 33054737	222	305	72.8	Clinical interview	37.5	38.4	European	EPIC	Whole blood
BiDirect	24924233	287	561	51.5	Questionnaire	51.6	49.7	European	EPIC	Whole blood
Dublin Cohort	26419460	37	186	19.9	Questionnaire	38.0	52.7	European	450K	PBMCs
Environmental Risk Longitudinal Twin Study	12236608; 12537874	322	1,625	19.8	Clinical interview	18.5	51.0	Predominantly European	450K	Whole blood
EXTEND	29790996	190	1,181	16.1	Self-reported diagnosis/GP visit	56.3	48.0	Predominantly European	450K	Whole blood
Finnish Twin Cohort (450K set)	31796134; 31640839	163	985	16.5	Clinical interview	23.8	45.9	Finnish, European	450K	Whole blood
Finnish Twin Cohort (EPIC set)	31796134; 31640839	93	366	25.4	Clinical interview	25.4	49.5	Finnish, European	EPIC	Whole blood
FOR2107	30267149	340	667	51.0	Clinical interview	35.3	36.7	European	EPIC	Whole blood
GS (sets 1 and 2)	30918249	1,632	9,502	17.2	Clinical interview	49.8	41.0	European	EPIC	Whole blood
Janssen	36319817	191	222	86.0	*DSM*-criteria questionnaire	42.8	19.7	Predominantly European	EPIC	Whole blood
Biopsychosocial factors of major depression in youth/University Hospital of Munich	30947532	381	633	60.2	Clinical interview	15.1	33.3	European	EPIC	Whole blood
Munich Antidepressant Response Signature/GlaxoSmithKline	18586274; 16822851	311	497	62.6	*DSM*-criteria questionnaire	48.1	42.5	European	450K	Whole blood
Netherlands Twin Register	31666148	436	2,701	16.1	Clinical interview	36.5	33.0	European	450K	Whole blood
Study of Health in Pomerania-Trend	35348705	84	492	17.1	*DSM*-criteria questionnaire	51.1	46.3	European	EPIC	Whole blood
TwinsUK	30760334	201	692	29.0	Clinical interview	59.0	0	European	450K	Whole blood
Understanding Society/UK Household Longitudinal Study Set 1	30401456	72	1,121	6.4	Self-reported diagnosis/GP visit	58.4	41.9	European	EPIC	Whole blood
Understanding Society/UK Household Longitudinal Study Set 2	30401456	277	2,361	11.7	Self-reported diagnosis/GP visit	51.1	45.9	European	EPIC	Whole blood

Mean ages are reported for two subsets of the Avon Longitudinal Study of Parents and Children cohort. *DSM*, *Diagnostic and Statistical Manual of Mental Disorders*^54^; GP, general practitioner; PMID, PubMed identifier.

**Table 2:** Significant CpG sites in the meta-analysis of MD MWAS

CpG	Chromosome	Base pair location	Beta	s.e.	*P*	*P*-adj	*N*	UCSC gene	Relation to Island	Array	EWAS catalog/EWAS atlas (PMID)	GWAS catalog gene look-up (PMID)
cg19432791	3	45894556	0.025	0.004	1.70 × 10^−9^	0.001	16,545	LZTFL1	OpenSea	EPIC	C-reactive protein (CRP)^a^ (38692281); glucose (33151971); schizophrenia (26619358)	Multiple sclerosis (31604244); COVID-19 (35910207); CD3 protein levels (32929287)
cg11101030	13	93744566	−0.035	0.006	4.99 × 10^−9^	0.004	15,901	–	OpenSea	EPIC	–	–
cg10145533	8	20350779	0.019	0.003	5.34 × 10^−9^	0.004	24,191	–	OpenSea	Both	Crohn’s disease^a^ (27279921); preterm birth^a^ (28428831); autoimmune disease^a^ (31024609)	–
cg12187985	18	23654410	0.021	0.004	5.43 × 10^−9^	0.004	16,850	SS18	OpenSea	EPIC	CRP^a^ (38692281); preterm birth (28428831); rheumatoid arthritis (23334450); LRIG1 protein (35945220)	Neuroticism (30595370); autoimmune thyroid disease (32581359)
cg09726355	17	42544621	0.019	0.003	8.53 × 10^−9^	0.007	16,628	GPATCH8	OpenSea	EPIC	Rheumatoid arthritis (23334450); HIV infection (27105112); alcohol consumption (27843151)	Cerebrospinal fluid progranulin levels (33087363); brain morphology (32665545); educational attainment (35361970)
cg03671627	2	209185667	0.026	0.005	9.85 × 10^−9^	0.008	16,850	PIKFYVE	OpenSea	EPIC	CRP^a^ (38692281); kidney disease^a^ (33933144); alcohol consumption (31789449); childhood obesity (28614626)	BMI (36581621); asthma (27611488)
cg17494199	13	106834942	−0.011	0.002	1.22 × 10^−8^	0.009	24,748	–	OpenSea	Both	Very early preterm birth^a^ (30833390), birth weight^a^ (31015461), fetal brain development^a^ (25650246)	–
cg17002328	14	91751773	0.016	0.003	2.32 × 10^−8^	0.018	24,750	CCDC88C	N_Shelf	Both	CRP^a^ (38692281); multiple sclerosis^a^ (30479356); chronic fatigue syndrome (30036399); schizophrenia (33279932); autism (27404287); BMI (33517419); ADHD (28785368)	White-matter hyperintensity volume (33293549); subcortical volume (32665545); Autism (35215271); Alzheimer’s disease (26830138)
cg12805451	3	123672734	0.024	0.004	3.11 × 10^−8^	0.024	16,850	CCDC14	OpenSea	EPIC	–	intelligence (29844566); general cognitive ability (29326435)
cg20793665	2	232549224	0.019	0.003	3.79 × 10^−8^	0.029	24,752	–	S_Shelf	Both	CRP^a^ (38692281)	–
cg01150646	17	1377304	0.013	0.002	3.80 × 1^−8^	0.029	16,850	MYO1C	S_Shore	EPIC	Insulin resistance (30792424); BMI (29278407); Alzheimer’s disease (33257653); alcohol consumption (27843151); preterm birth (28428831)	Alzheimer’s disease (35770850); educational attainment (30038396)
cg02948555	15	42749848	0.036	0.007	4.72 × 10^−8^	0.036	15,911	ZNF106	OpenSea	EPIC	Downs syndrome (33547282); early onset intracranial atherosclerotic stenosis (31142690)	CRP (31900758); cholesterol to total lipid ratio in low-density lipoprotein cholesterol (35213538)
cg10604091	13	50650086	0.023	0.004	5.27 × 10^−8^	0.040	16,850	DLEU2	OpenSea	EPIC	BMI (29278407); inflammatory bowel disease (32281463); Alzheimer’s disease (25129075); birth weight (31015461); insufficient sleep (30718923)	BMI (31669095); creatinine (35710981, 34594039); non-high-density lipoprotein cholesterol levels (34887591)
cg04173586	19	2167496	0.025	0.005	6.30 × 10^−8^	0.048	24,043	DOT1L	S_Shore	Both	Schizophrenia^a^ (33646943); alcohol consumption^a^ (31789449)	BMI-adjusted waist/hip ratio (30575882); gamma-linolenic acid (26584805)
cg07063853	11	43333512	0.020	0.004	6.42 × 10^−8^	0.049	23,395	API5	Island	Both	HIV infection^a^ (27105112); obesity (29692867); rheumatoid arthritis (27585642); Alzheimer’s disease (33257653)	–

EWAS catalog (http://www.ewascatalog.org/), EWAS atlas (https://ngdc.cncb.ac.cn/ewas/atlas) and GWAS catalog (https://www.ebi.ac.uk/gwas/) were used to find associated traits in previous studies (search was based on genes). Beta, standardized regression coefficient; *P*, double-sided *P* value; *P*-adj, Bonferroni-corrected *P* value.

^a^Traits associated with CpG sites.

Five of the significant CpG sites positionally mapped to genes associated with mental health, neurodegenerative and developmental disorders. The gene mapped from cg17002328 (*CCDC88C*) was associated with schizophrenia^17^ and attention-deficit/hyperactivity disorder (ADHD)^16^ in epigenetic studies and with brain structural measures in GWAS, such as cortical surface area^18^ and accumbens volume^19^. CpG site cg17494199 was associated with preterm birth^20^, birth weight^21^ and fetal brain development^22^ in previous epigenetic studies. Genes mapped from cg01150646 (*MYO1C*), cg10604091 (*DLEU2*) and cg07063853 (*API5*) were associated with Alzheimer’s disease in brain-tissue methylation levels^23–25^.

A total of five significant CpG sites were associated with autoimmune diseases and biomarkers in previous studies, one of which was the most significantly CpG site (cg19432791 on chromosome 3), mapped to gene *LZTFL1*. From the catalog of epigenome-wide association studies (EWAS catalog), the *LZTFL1* gene was associated with biomarkers related to pain, such as glucose level^26^, and autoimmune diseases or markers, such as rheumatoid arthritis^27^ and C9 protein levels^28^. Four of the significant CpG sites, cg12187985 (*SS18*), cg10145533, cg09726355 (*GPATCH8*) and cg07063853 (*API5*), map to genes previously found associated with autoimmune markers and diseases (for example, rheumatoid arthritis^27^) in MWAS studies.

Other CpG sites, cg03671627 and cg02948555 (*ZNF106*), were associated with traits and markers relevant to obesity in both genetic and epigenetic studies. These markers include, for example, BMI^29^ and the ratio of cholesterol to total lipids^30^.

A complete list of CpG sites and related genes and traits can be found in Table 2. Quantile–quantile plot of the meta-analysis and the inflation factors of the association statistics for each individual cohort can be found in Supplementary Figs. 3 and 4, respectively. See Supplementary Fig. 5 for effect sizes of each study for the significant CpG sites.

### Identification of DMRs

A total of 37 differentially methylated regions (DMRs) were identified to be associated with MD after Bonferroni correction. The largest associated region locates within the major histocompatibility complex region, discoidin domain receptor tyrosine kinase 1 (*DDR1*) gene (chr. 6:30853258–30854233), previously implicated in major psychiatric disorders (for example, schizophrenia, MD and bipolar disorder).

The full list of significant DMRs can be found in Supplementary Table 1.

### Pathway enrichment analysis

Following the meta-MWAS, pathway enrichment analysis was conducted to identify potential biological pathways that the significant CpG sites were implicated in. No gene ontology (GO) term or Kyoto Encyclopedia of Genes and Genomes (KEGG) pathway was significantly enriched after false discovery rate (FDR) correction (Supplementary Table 2). The top ten most significantly enriched GO terms included pathways relevant to protein and metabolic processes (for example, negative regulation of protein localization to ciliary membrane) (*P* ranged from 0.003 to 8.14 × 10^−4^). For KEGG, ‘transcriptional misregulation in cancer’ and ‘lysine degradation’ were the only pathways that reached nominal significance (*P* ≤ 0.007).

### The basic model versus complex model

**Fig. 2:**
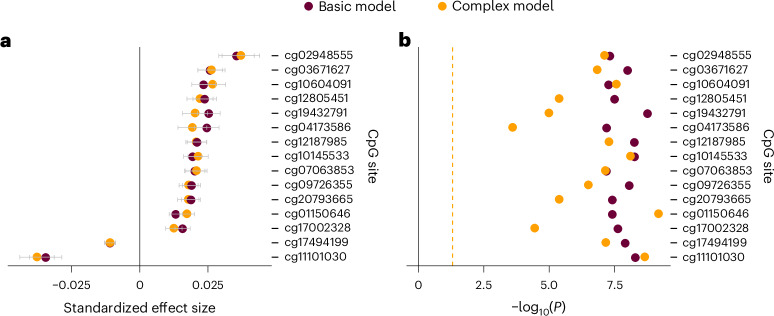
Comparison between basic and complex models. **a**,**b**, In both comparison of effect sizes indicated by standardized coefficients of linear regression (**a**) and comparison of double-sided *P* values (**b**), each dot represents a CpG probe. In **a**, the *x* axis represents effect size, and in **b**, the *x* axis represents −log_10_-transformed, double-sided *P* value. Error bars represent ± one standard error of the mean. Differences in *P* values reflect both the model used and the sample sizes. The *y* axis represents individual CpG sites. The yellow dashed line in **b** represents the significance threshold for nominal significance (double-sided *P* < 0.05).

We compared results for the basic model (18 cohorts, *N*_total_ = 24,754) and complex model (15 cohorts, *N*_total_ = 20,196; Supplementary Fig. 1) to evaluate potential confounding effects of BMI and alcohol consumption (Fig. 2). Effect sizes of the two models were highly correlated for the significant CpG sites (*r* = 0.988) and for CpG sites across the entire methylome (*r* = 0.920). All significant CpG sites in the basic model remained significant in the fully adjusted model after Bonferroni correction across the 15 significant CpGs found in the basic model, despite a significant reduction in sample size (*P* < 2.51 × 10^−4^, *P*_Bonferroni_ < 0.004 corrected across the significant CpG sites in discovery analysis).

### Out-of-sample classification of MD using methylation scores

**Fig. 3:**
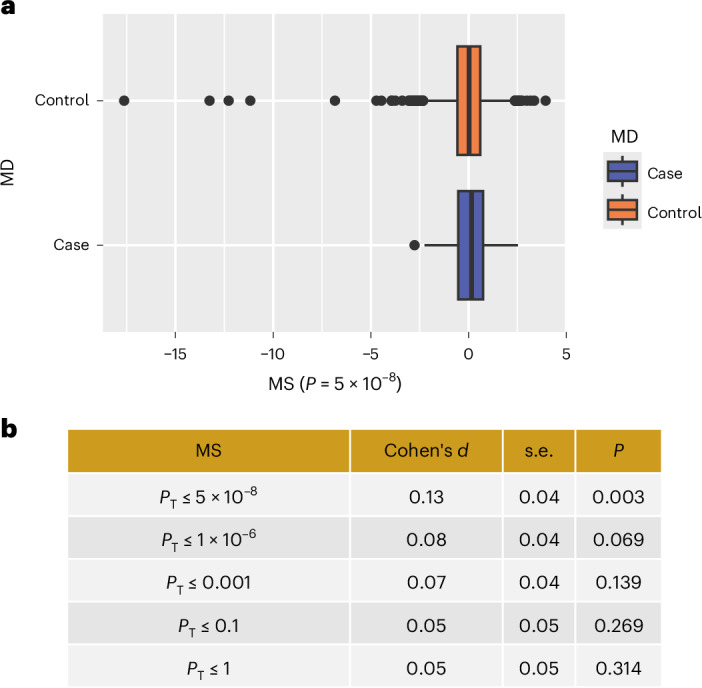
Out-of-sample classification of MD using MS. **a**, Box plot for case–control classification using MS created using *P*-value threshold at 5 × 10^−8^. MS was standardized and residualized against age, sex and values for aryl hydrocarbon receptor repressor (AHRR) probes. Residuals of MSs were used for the plot. The *x* axis represents MS, and the *y* axis represents MD case and control groups. Box boundaries are determined by the top and bottom quartiles. Box whiskers are defined by 1.5 interquartile range. Any values outside of the box whiskers are presented as individual data points. Median values per group are indicated as vertical lines within the boxes. **b**, Statistics for MS association tests. Cohen’s *d* was calculated by comparing MD case–control differences in MS. Double-sided *P* values are reported. *P*_T_, *P* threshold for creating the MS.

MD methylation scores (MD–MSs) were used to assess whether the present MD MWAS summary statistics can be used to classify MD case–control status in an independent sample. All five MD–MSs showed positive effect sizes (higher score associated with higher liability of MD). There was an increasing trend of effect sizes as the *P*-value threshold for MS calculation became increasingly stringent (Fig. 3). Of the five scores tested, only MS at *P*-value threshold of ≤5 × 10^−8^ was found to be associated with MD diagnosis (*β* = 0.13, *P* = 0.003, area under the curve = 0.53; Fig. 3). *P* values for other MSs ranged from 0.069 to 0.314.

### Association between MD–MS and inflammatory protein abundance

Following out-of-sample classification using MD–MS, we sought to identify inflammatory proteins of which the abundance was associated with MD–MS. Five proteins were significantly (*P* < 3.5 × 10^−3^) associated with the MD–MS created at the *P* threshold of ≤5 × 10^−8^. The strongest association was found in tumor necrosis factor B (*β* = −0.15, *P* = 2.37 × 10^−5^). Other proteins were interleukin 6 (*β* = 0.12, *P* = 5.18 × 10^−4^), transforming growth factor alpha (*β* = 0.11, *P* = 2.8 × 10^−3^), CD5 (*β* = −0.11, *P* = 2.1 × 10^−3^) and EN-RAGE (*β* = 0.11, *P* = 3.5 × 10^−3^; Supplementary Fig. 6).

### Heterogeneity analysis

#### Leave-one-out analysis

We systematically investigated the heterogeneity of effect sizes in each individual study by conducting leave-one-out (LOO) analysis. For every CpG site, all effect sizes for leave-one-out analyses remained in the same direction as the meta-analysis (Supplementary Fig. 7). All tested CpG sites remained significant after leaving individual studies out except when the largest study, Generation Scotland (GS), was left out (*P* < 1.03 × 10^−4^). When GS was left out, 11 out of the 15 tested CpG sites remained nominally significant (*P* < 8.91 × 10^−3^). The four sites that became non-significant when GS was omitted were cg02948555 (*β*_meta-MWAS_ = 0.036, *β*_LOO_ = 0.008, *P*_LOO_ = 0.511), cg11101030 (*β*_meta-MWAS_ = −0.035, *β*_LOO_ = −0.01, *P*_LOO_ = 0.235), cg07063853 (*β*_meta-MWAS_ = 0.02, *β*_LOO_ = 0.01, *P*_LOO_ = 0.232) and cg01150646 (*β*_meta-MWAS_ = 0.013, *β*_LOO_ = 0.007, *P*_LOO_ = 0.054).

#### Effect of age difference between studies

Due to systematic differences of age range across studies, we conducted meta-regression to investigate the impact of age difference on the findings of meta-analysis. Of the 15 CpG sites significant in the meta-MWAS, 14 sites did not show an effect of age difference across studies (absolute *β* ranged from 1.11 × 10^−6^ to 6.09 × 10^−4^, *P* > 0.234; Supplementary Fig. 8). One site, cg04173586, showed a significant effect of age (*β* = −9.25 × 10^−4^, *P* = 0.006). However, leave-one-out analysis showed highly consistent findings for cg04173586 across studies. There were 14 out of 17 participating cohorts that showed effect sizes consistent with the meta-MWAS (Supplementary Fig. 5) and leave-one-out analyses were significant for all iterations (*P* ranged from 1.03 × 10^−4^ to 2.38 × 10^−8^; Supplementary Fig. 5 and Supplementary Table 3).

#### Correlation matrix for effect sizes

Heterogeneity between studies was analyzed by looking at the between-study correlation of effect sizes estimated using the basic model (Supplementary Fig. 9). Correlations between the 18 studies participating in the MD meta-MWAS ranged from −0.19 to 0.31. The highest positive correlation was found between the Netherlands Twin Register and Janssen (*r* = 0.31). Of the 153 pairwise correlations, 96 were positive (62.7%). Compared with the MD meta-MWAS, BMI MWAS (10 studies) showed higher, positive effect size correlations between studies (*r* ranged from 0.305 to 0.864, 100% of the pairs were positively and significantly correlated).

### Mendelian randomization

**Fig. 4:**
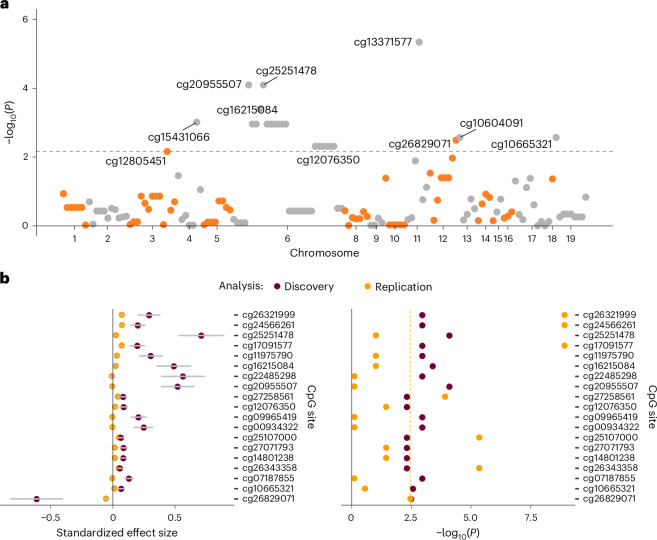
MR analysis of the causal effect of DNAm on MD. **a**, *P* plot for discovery MR analysis in GS. Each dot represents a CpG site. The *x* axis represents chromosomes and base pair position. The *y* axis represents −log_10_-tranformed *P* value of Wald ratio MR analysis. The gray dashed line shows the FDR-corrected significance threshold. Discovery MR analysis was performed on CpG sites available in the EPIC array. **b**, Replication analysis in GoDMC for the significant CpGs found in the discovery analysis. Replication analysis was performed on CpG sites available on the 450K array. Of the 23 significant CpG sites in the discovery analysis, 19 were available on both EPIC and 450K arrays and therefore were included in the replication analysis. The *x* axes represent effect size and −log_10_-transformed *P* value on the left and right panels. The *y* axis represents individual CpG sites. The yellow dashed line in the right panel represents FDR-significance threshold.

Using *cis* mQTL data from GS and Wald ratio Mendelian randomization (MR) method, we found 23 significant and potentially causal effects of DNA methylation on MD (absolute *β* ranged from 0.06 to 0.93, *P* ranged from 6.88 × 10^−3^ to 4.58 × 10^−6^; Fig. 4 and Supplementary Data 1). There were 17 CpG sites located in the major histocompatibility complex region (mapping to the *DDR1R* gene) on liability to MD (*β* ranged from 0.06 to 0.17, *P* ranged from 1.1 × 10^−3^ to 8.05 × 10^−5^). See Supplementary Data 1 for the full list of significant causal effects found in the discovery analysis in GS.

Of the 23 potentially causal effects found in the discovery analysis, 4 CpG sites were specific to the EPIC array, while 19 were available on both array types and could, therefore, be included in the replication analysis. Seven CpG sites tested were replicated (*P* < 0.003; Fig. 4). Six of the replicated CpG sites were in the major histocompatibility complex region (*β* 0.040 to 0.073, *P* 1.22 × 10^−4^ to 1.19 × 10^−9^). In addition, a potentially causal effect from cg26829071 to MD (mapping to *GPR133*, *β* = −0.056, *P* = 0.004) was found on chromosome 12.

No significant causal effects from MD to DNAm were found (absolute *β* 1.62 × 10^−5^ to 0.22, *P*_uncorrected_ > 0.001, *P*_FDR_ > 0.66).

### MWAS in East Asian ancestry

We sought to identify DNAm associations with MD in East Asian ancestry using data from Taiwan Biobank. There were no methylome-wide significant findings identified. Correlation between the methylome-wide significant CpGs effect sizes identified in the main results (*N* = 15) and the same CpGs in Taiwan Biobank was *r* = 0.48. The effect direction was consistent between the two cohorts for 11 of the 15 CpGs (73.3% in the same direction; Supplementary Tables 4–6). Effect sizes were significantly and positively correlated when we extended the comparison in the top 100 and top 1,000 CpG sites found within the MWAS from European samples (top 100 CpG sites found in European samples: *r* = 0.26, *P* = 0.009; top 1,000 CpG sites found in European samples: *r* = 0.22, *P* = 4.07 × 10^−12^).

## Discussion

Our meta-MWAS of 24,754 individuals found 15 CpG sites to be significantly associated with MD, an increase of 12 CpGs on previously reported associations^11^. Significant findings revealed CpGs mapped to genes associated with autoimmune markers and depression-related traits, such as BMI. Seven potentially causal effects from DNAm to MD were identified and replicated. Leave-one-out analysis showed that effects were highly consistent across studies for the significant sites. However, the correlation in CpG–MD association effect sizes was relatively low and showed considerable between-study heterogeneity for MD, in contrast to BMI, which showed uniformly positive and more homogeneous correlations across all available studies. A positive correlation was observed for top MD CpG sites between European and East Asian samples (*r* = 0.48 for significant CpG sites and *r* = 0.26 for top 100 CpG sites), and effects of 11 of the 15 significant CpG sites found in the European sample remained in the same direction in the East Asian sample.

Five CpG sites mapped to genes previously reported in association with autoimmune disorders and markers relevant to activity of the hypothalamic–pituitary–adrenal (HPA) axis. The top CpG, cg19432791, mapped to *LZTFL1*, a gene that was identified as an effector gene that contributes to severe autoimmune responses and inflammation, such as risk of respiratory failure caused by COVID-19^31^. In addition to its association with inflammation, *LZTFL1* regulates ciliary localization of the Bardet–Biedl syndrome protein complex. The Bardet–Biedl syndrome complex is a well-replicated causative protein marker for obesity^32^ and highly relevant to HPA-axis activity by being involved in transducing leptin signals in hypothalamic neurons^33,34^. Several top CpG sites and mapped genes in our MWAS were previously linked to other psychiatric disorders. For example, two CpG sites, cg04173586 and cg19432791, were associated with schizophrenia^35,36^. Another CpG site, cg17002328, mapped to gene *CCDC88C*, was found to be related to multiple psychiatric disorders, including schizophrenia^17^, attention-deficit/hyperactivity disorder^16^ and autism^37^. Four of the 11 genes identified in our MWAS (*LZFL1, SS18, CCDC88C* and *DLEU2*) were found in significant DMRs associated with suicide attempts in adults with bipolar disorder^38^. Beyond psychiatric disorders, five CpG sites showed associations with CRP^8,39^. CRP is one of the most well-studied predictors of vulnerability to depression^40^ and persistence of depressive symptoms^41^. As a pro-inflammatory cytokine, CRP has been associated with stress-related hyperactivity in the HPA axis^42^. The proteome-wide association analysis study (PWAS) findings provided additional evidence for the association between the top CpG sites and inflammation, for example, in tumor necrosis factor beta and interleukin 6 (IL6). Our findings provide further evidence supporting the association between MD and chronic inflammation, particularly involving the HPA axis. However, no association between these results and MD had been identified in previous MWAS, probably due to the limited sample sizes of MWAS conducted on mental health-related traits.

Three CpG sites were associated with traits (for example, blood cholesterol levels, waist-to-hip ratio and pulse pressure) related to BMI. Obesity has been repeatedly associated with depression, and MR analyses from previous studies have indicated that this may be a causal association^3,43^. Within the genes in the vicinity of the four CpG sites, *ZNF106* is involved in the regulation of calcium homeostasis that is crucial for cell survival and death^44^. Other genes, including *MYO1C* and *FNDC3B*, are understood to be involved in energy metabolism and homeostasis and highly sensitive to stress and inflammation^45,46^. Although the previous findings relevant to the BMI-related genes were based on GWAS studies, one of the genes, *FNDC3B*, was found in an earlier MD MWAS study based on brain-tissue samples^13^. Considering the dynamic nature of DNA methylation and its high sensitivity to environmental stressors, our findings suggest metabolic processes may play a potentially crucial role in depression, which may be exacerbated by adverse environmental factors and dysfunctional stress coping mechanisms.

We found stronger evidence for DNAm causing MD than for the reverse direction. This aligns with the broader investigation by Min et al., which showed that DNAm more frequently affects complex traits than vice versa^15^. However, previous research has shown MD can influence DNAm at CpG sites linked to MD genetic risk loci^47^. While our MWAS identified a dominant causal pathway from DNAm to MD in top CpG sites identified in our MWAS, reverse causation may occur in other genomic regions, particularly at MD genetic risk loci^47^.

We observed varying results from different cohorts in our study, with little evidence of systematic differences in study age ranges contributing to the heterogeneity. The higher degree of effect size heterogeneity in the MD versus BMI MWAS meta-analyses suggests that phenotype may be a reason for differences between the studies and not other methodological factors, such as sample processing or covariate adjustment. In the MD meta-analysis, larger studies (*N* > 1,000) showed stronger correlation for the top associations, suggesting that sufficient statistical power may help overcome the issue of phenotyping inconsistency. This suggests that future individual MWAS studies of MD should be larger. The considerable costs of DNAm profiling, compared with genotyping, act as a barrier to achieving these aims.

Although there was no significant finding in the MWAS conducted in non-European studies, it is notable that effects were consistent with the significant findings on European samples. Top pathways associated with MD also showed convergent results with the main analysis (for example pathways relevant to inflammation and immune processing). Correlations for effect sizes between European and East Asian samples were positive with the South Asian sample, consistent with previous evidence showing relatively high genetic consistency between European and East Asian groups^48^. Developing larger non-European DNA methylation samples will be crucial to provide a statistical balanced comparison between ancestry groups and to identify ancestry-group-specific DNA methylation sites for MD^49^.

Our study combined many studies with widely varying sample sizes, compared results between two ancestry groups and replicated MR findings using two large mQTL datasets. We provided a comprehensive evaluation of sampling and analytic strategies to guide future large-scale meta-MWAS for mental health disorders. While the blood draw was not timed to coincide with the onset of a depressive episode, limiting causal inferences based on the temporal order of DNAm exposure and MD onset, the MR analyses helped to address this potential limitation. Further, we appreciate that factors, such as antidepressant use, could potentially influence the relationships observed^50^. Future research should consider environmental factors as potential moderating factors to better understand the epigenetic variation in individuals with MD. In addition, there is a lack of replication in other tissue types that are directly relevant to mood regulation, such as brain tissue. Studies have shown that the genetic drivers of DNAm have similar effects across multiple cell types^51^. Future clinical applications and larger sample sizes make whole-blood DNAm data more feasible than post-mortem tissue samples. However, to ensure the validity of the findings, future studies should broaden their scope by encompassing additional cell and tissue types.

## Methods

### Participants

A total of 24,754 European-ancestry participants (5,443 MD cases) from 18 studies were included in the meta-analysis. The mean age of participants in each study ranged from 15 to 59 years. Details for each individual study can be found in Table 1 and Supplementary Information, ‘Cohort details’ section. Written consent was obtained from all participants. The study was approved by the NHS Tayside Research Ethics committee (05/s1401/89). Ethics declaration and participant compensation for each participating cohort can be found in the Supplementary Information, ‘Cohort details’ section.

### DNAm data preparation and quality check

DNAm data were obtained from DNA extracted from whole blood. Eight studies used the Infinium Human Methylation 450 (450K) BeadChip array (Illumina Inc.; number of CpG sites ranged from 275,868 to 438,752 after quality check), and the other ten studies used the Illumina Infinium Methylation EPIC array (Illumina Inc., number of CpG sites ranged from 673,085 to 809,447 after quality check). Quality checks and data normalization were conducted by each individual study team. Details are provided in the protocol papers for each individual study (Table 1 and Supplementary Information, ‘Cohort details’ section). In brief, the majority of studies used functional normalization for methylation data preprocessing, unless stated otherwise in the Supplementary Information, ‘Cohort details’ section^52^. Similar quality check procedures were used, including removing probes with outlying signal intensity, bead count and detection *P* values, removing participants with mismatched sex prediction from DNA methylation data, and removing cross-hybridizing probes that map to common genetic variants (at minor allele frequency > 0.05) or polymorphic probes^52^. *M* values were used for the association analysis^53^.

### Diagnosis of MD

Lifetime diagnosis of MD was derived on the basis of structured clinical interview or self-reported symptoms. Those studies that derived diagnoses of MD on the basis of structured clinical interviews used criteria from the *DSM* Fifth/Fourth Edition^54^. Self-declared MD was based on depressive symptoms lasting for more than 2 weeks and help-seeking due to depression. Studies that derived MD diagnosis on the basis of multiple time points defined cases as those who experienced any depressive episodes during their lifetime, and controls were those who did not declare MD throughout. A total of 9 studies defined MD cases using structural clinical interview (*N* cases = 3,790), 3 studies used *DSM*-criteria questionnaires (*N* cases = 586), 3 studies used self-administered questionnaires for depressive symptoms (*N* cases = 528), and 3 studies defined MD cases on the basis of self-declared visits to a general practitioner (*N* cases = 539). Details for MD diagnosis for each cohort can be found in Table 1 and the Supplementary Information, ‘Cohort details’.

Additional exclusion criteria per study are stated in the Supplementary Information, ‘Cohort details’ section.

### Association analysis

MWAS was conducted in each individual study before the meta-analysis. Linear regression was used to test the association between DNAm (*M* values, dependent variable) and MD diagnosis (independent variable) using a pipeline available at the URL https://github.com/psychiatric-genomics-consortium/mdd-mwas. Those cohorts that used their own specific pipelines are specified in the Supplementary Information, ‘Cohort details’ section. The pipeline uses the R package ‘limma’ (version 3.60.6) for linear regression on large omic data^55^. Three models representing increasingly rigorous correction for potential confounders were estimated. Covariates for the simplest model were age, sex, batch, the first 20 methylation principal components (PCs) or surrogate variables^56^ based on the study protocol for each individual cohort, and white-blood-cell proportions estimated from DNAm data of CD8^+^ T cells, CD4^+^ T cells, natural killer cells, B cells and granulocytes^56^. The AHRR-adjusted model had an additional covariate that adjusted for smoking status by including the *M* values for the AHRR probe (cg05575921), due to its known accuracy in predicting smoking^57^ and its consistent availability in all studies. Finally, a third model with additional covariates (referred to as the ‘complex model’) was fitted that contained BMI and alcohol consumption in addition to all the other covariates included in the previous models.

Results for the AHRR-adjusted model (referred to as the ‘main model’) are reported as the main findings. Standardized Cohen’s *d* between MD cases and controls were reported as effect sizes.

### Meta-analysis

Meta-analysis of cohort-level MWAS was conducted using METAL (version 2011)^58^ in a two-stage process. First, meta-analysis was performed on studies that used 450K and EPIC arrays separately, due to the difference in CpG sites available for each array (Fig. 1).

Those CpG sites that either were available for more than half of the studies using the given array or had a total sample size over 80% of the max sample size were kept for further analysis. CpG sites with excessive standard errors (s.e. > 0.5; Supplementary Fig. 1) were removed from analysis. Second, the summary statistics for 450K and EPIC array were meta-analyzed. A fixed-effect inverse-variance model was used without genomic control correction. *P* values were Bonferroni corrected (*P*-value threshold = 6.55 × 10^−8^ to reach Bonferroni-corrected significance) for all 763,265 CpGs included in the analysis. CpGs were mapped to genes using an annotation object generated by the ‘IlluminaHumanMethylationEPICanno.ilm10b4.hg19’ R package (version 3.13)^59^. The annotation object was created on the basis of the product file provided by Illumina for the Infinium MethylationEPIC v1.0 Beadchip^60^, with the UCSC gene names provided as the target gene regions of the assay. We searched the EWAS Atlas (https://ngdc.cncb.ac.cn/ewas/atlas) and EWAS Catalog (http://www.ewascatalog.org/) for significantly associated CpGs and genes and the GWAS Catalog (https://www.ebi.ac.uk/gwas/) for annotated genes.

### Pathway enrichment analysis

Following the meta-MWAS, we conducted pathway enrichment analysis to identify the biological pathways in which the significant CpG sites may be implicated. We used the ‘gometh’ function from the ‘missMethyl’ R package (version 1.38.0)^61^ for pathway analyses using the results of the AHRR-adjusted model. Significant CpGs found in the MWAS after Bonferroni correction were selected, and the rest of the CpGs profiled in the EPIC array were included as the background list. GO terms and KEGG pathways were analyzed separately. *P* values for both enrichment analyses were FDR corrected.

### Identification of differentially methylated regions

DMRs associated with MD were identified using the ‘dmrff’ R package (version 1.1.2)^62^. A DMR was identified if it contained at least two CpG sites within a maximum of 500-bp window, showed consistent direction of effect and both/all had meta-MWAS *P* < 0.05. Statistics were meta-analyzed within the identified region. *P* values were Bonferroni corrected for all regions (>2 CpG sites) and single CpG sites altogether. Significant DMRs were identified if the two-sided, Bonferroni-corrected *P* < 0.05.

### Analysis of the confounding effect of BMI and alcohol consumption

BMI and alcohol consumption are risk factors for MD and are known to have widespread associations with DNAm^7,29^. To investigate whether the signals found in the main model were due to the effects of BMI and alcohol consumption, we conducted an additional meta-analysis of a complex model for the 14 cohorts that had BMI and alcohol consumption data available (*N*_total_ = 20,196; see Supplementary Fig. 2 for sample sizes of individual studies). BMI and alcohol consumption were added as additional covariates for the complex model. We compared effect sizes and *P* values between the basic and complex models for significant associations found in the main model.

### Out-of-sample classification of MD using MD–MS

#### Calculation of MD–MS and statistical model

We created MD–MS in an independent testing sample by calculating the weighted sum of *M* values. Effect sizes from the MD meta-MWAS summary statistics were used as weights. Five *P*-value thresholds were used to select the CpG sites—*P* ≤ 1, *P* ≤ 0.01, *P* ≤ 0.001, *P* ≤1 × 10^−6^ and *P* ≤ 5 × 10^−8^—resulting in five MD–MSs.

MD diagnosis was set as the independent variable and MS as the dependent variable in the logistic regression model using the ‘glm’ function in R. Covariates were age, sex and *M* values of AHRR probe. DNAm-estimated cell proportions were not associated with any of the MD–MSs (*P* > 0.5) and were therefore not included as covariates. Batch and genomic relationship matrix were pre-corrected by residualizing *M* values against these covariates.

#### Testing sample

GS DNAm set 3 data were used for out-of-sample classification (GS DNAm sets 1 and 2 were included in the meta-MWAS). GS DNAm set 3 data used the Illumina Infinium Methylation EPIC array, had no overlap with the GS DNAm data used in the meta-MWAS, and relatedness within set 3 and with the rest of the GS sample was removed by regressing the *M* values against the genomic relationship matrix. Quality check and preprocessing were kept consistent with the GS sample used in the meta-MWAS (sets 1 and 2).

MD diagnosis was derived using electronic health records (EHRs) from GP diagnosis^63^. Details for the EHRs were explained in the protocol paper by Kerr et al.^63^. In brief, a subset of participants (*N* = 20,032) of GS with genotyping data gave consent to link their data to EHRs. All Read codes from the PH1021^64–66^ and PH149^67,68^ inventories of the Health Data Research UK Phenotype Library for primary care data of depression were used to identify cases of lifetime MD. Participants with ≥1 entry of diagnosis of depression were classified as cases, and those with no entry of any diagnosis for depression or no data to indicate depressive status were controls. The final testing sample contained 504 cases and 8,372 controls.

### Association between MD–MS and inflammatory protein markers

A previous study demonstrated widespread association between protein abundance and DNAm^28^. We conducted a PWAS for MD–MS seeking to reveal the potentially downstream proteomic consequences of the measured DNAm differences.

Lothian Birth Cohort 1936 (LBC1936), a cohort independent of the MD MWAS, was used for the PWAS analysis. LBC1936 is a community-based birth cohort of participants born in 1936, recruited in Scotland. The sample used in the PWAS analysis had 875 people with both DNAm and proteome data collected at mean age 69.8 ± 0.8 years. DNAm in LBC1936 was profiled in whole-blood samples using the HumanMethylation450 BeadChip Kit (Ilumina). Sample preparation and quality check were kept consistent with previously published work^69^. Proteomic data were profiled using lithium heparin collected plasma samples analyzed using a 92-plex proximity extension assay (inflammation panel; Olink Bioscience). The Olink team performed preprocessing using NPX Manager software. For 22 proteins, over 40% of samples fell below the lowest limit of detection, leaving 70 post-quality-check proteins.

General linear models were used to test the association between relative abundance across all 70 proteins and MD–MS created at *P* threshold ≤ 5 × 10^−8^. Protein abundance levels were rank-based-inverse normalized and residualized against age, sex, first four genetic PCs and array for proteomic data before entering association analysis. Residual scores of protein abundance were set as the dependent variable. Array for DNAm data and the AHRR probe were included as covariates in the GLM. Two-sided *P* values were corrected using FDR correction.

### Heterogeneity analysis

#### Leave-one-out analysis

To investigate whether a particular study had disproportionate influence on any meta-analytic association, we conducted leave-one-out meta-analyses. For each significant CpG, 18 iterations of meta-analysis were conducted leaving each individual study out, regardless of profiling arrays.

#### Meta-regression for age

For the CpGs that were significant in the MWAS meta-analysis, we used meta-regression to analyze the potential effect of age across studies. A mixed-effect model with the ‘metareg’ R package^70^ was used for the meta-regression analysis. Mean age of each individual cohort was set as a random effect. Standardized regression coefficients from summary statistics of each individual study were set as estimates of treatment effects (the ‘TE’ option). Between-study variance was estimated using the restricted maximum likelihood method. The standardized regression coefficient of the random effect of age was reported as effect sizes for the meta-regression analysis.

#### Comparison between MD and BMI associations

To further evaluate the heterogeneity of MD–DNAm associations between studies, we looked at the correlation of effect sizes for the summary statistics of the MD MWAS for individual studies. GS that was included in the MWAS, being the largest study sample in the meta-analysis (*N* = 9,502), was used to select a list of CpG sites of interest. The 1,000 most significantly MD-associated CpG sites in GS were selected. Effect sizes for these CpG sites were extracted for all other studies. Correlation analysis was conducted on the effect sizes. We also performed an MWAS of BMI and conducted a similar analysis, for comparison with MD, to assess whether BMI–DNAm associations were similarly heterogeneous.

### MR

To identify potentially causal CpG sites to MD, we conducted MR analyses using Genetics of DNA Methylation Consortium (GoDMC; discovery analysis) and GS data (replication analysis).

#### MD GWAS summary statistics

GWAS summary statistics were obtained from the Howard et al. meta-analysis for MD GWAS from the PGC, 23andMe and UK Biobank^3^. A total of 807,553 individuals (246,363 cases and 561,190 controls) of European ancestry were included in the MD meta-GWAS.

#### GoDMC and GS methylation quantitative trait loci

Quantitative trait loci associated with DNAm (mQTL) summary statistics were obtained from GS and GoDMC. For GS, full mQTL summary statistics (*N* = 17,620) were obtained without any *P*-value thresholding. OmicS-data-based complex trait analysis was used for mQTL estimation^47,71^. Covariates were kept consistent with the main model for the MD MWAS, except for using self-reported smoking behavior (current smoker, past smoker or non-smoker) and pack years (quantity of smoking) to control for smoking and adding ten genetic PCs as covariates. GoDMC mQTL data were obtained through the consortium website (http://www.godmc.org.uk/)^15,47^ using the same pipeline described in the GoDMC protocol paper by Min et al.^15^. The mQTL data contains 32 cohorts with 25,561 participants of European ancestry. The mQTL meta-analysis from GoDMC adopted a two-stage approach. First, a truncated set of mQTL data that reached the threshold of *P* value < 1 × 10^−5^ were obtained from participating cohorts. This initial stage created a candidate list of mQTL associations (*n* = 120,212,413). Meta-analyses for mQTL were then conducted on these candidate associations.

Samples for GS mQTL analysis, GoDMC mQTL analysis and MD GWAS were mutually exclusive.

#### Selection of CpG list

A list of CpG sites that were either (1) significant in the MD MWAS or (2) within the identified DMRs were selected as CpG sites of interest. We extracted *cis* mQTL for further MR analysis^15^.

In the GoDMC dataset, a total of 156 CpG sites that met the preceding criteria had at least one *cis* mQTL. The *cis* mQTL summary statistics for these CpG sites were extracted from the GoDMC dataset. For those CpG sites that had more than one *cis* mQTL after clumping (1 Mb window, *P* < 5 × 10^−8^), the most significant mQTL with the lowest *P* value was selected for analysis. Those CpG sites that showed significant causal association with MD were selected for replication analysis using the GS mQTL summary statistics.

#### MR methods

MR analysis was conducted using the ‘TwoSampleMR’ R package (version 0.5.6)^72^. To identify causal effects of DNAm on MD, we used the Wald ratio MR method^73^ to analyze causal effects on MD using *cis* mQTLs (within a 1 Mb window in vicinity of the chosen CpG site). The most significant mQTL for each CpG site that reached the threshold of *P* < 5 × 10^−8^ was selected. Causal effects from DNAm to MD were tested using both GoDMC and GS mQTL data. For the causal effect in the reverse direction (from MD to DNAm), MD GWAS summary statistics were clumped at *P* ≤ 5 × 10^−8^, with a 1 Mb window and *r*^2^ = 0.001. Causal effects from MD to DNAm were tested using mQTL data from the entire GS sample (sets 1, 2 and 3).

### MWAS in East Asian ancestry

We sought to investigate MD associations with DNAm in participants of East Asian (Taiwan Biobank) ancestry. Demographic and descriptive statistics are included in the Supplementary Information, ‘Cohort details’ section. As in the main meta-analysis, biological and technical covariates, as well as age, sex and smoking (indexed by AHRR probe cg05575921), were included as covariates. Evidence of trans-ancestry transferability of MD CpG effects was investigated by testing for the correlation of effect sizes across both ancestries. We then used the function ‘gometh’ in package ‘missMethyl’ to assess ontology and pathway enrichment (GO and KEGG) for differentially methylated CpG sites at a threshold of *P* < 1 × 10^−5^ (*N*_CpG_ = 24), as used in previous studies^74^.

### Reporting summary

Further information on research design is available in the Nature Portfolio Reporting Summary linked to this article.

## Supplementary information

Supplementary Information Supplementary Figs. 1–9, Tables 1–6, Methods, Results and legend for Supplementary Data 1.

Reporting Summary

Supplementary Data 1 Results for Mendelian Randomization (MR) analyses on causal effect of DNAm to MD. The table consists of results for discovery MR in Generation Scotland and replication MR using mQTL data from GoDMC. Results for Wald’s ratio tests are shown: beta, Wald’s ratio effect size; SE, standard error; pval, double-side *P* value for Wald’s ratio tests; pFDR, FDR-corrected *P* value. Following the Wald’s ratio results is information for the genetic instruments used for Wald’s ratio analysis (SNP info),: SNP, rsID of instrument; CHR, chromosome; BP, base pair position; A1, reference allele; A2, alternative allele; Freq, allele frequency estimated from the MD GWAS. In addition, effect sizes (beta), standard errors (SE) and *P* values (pval) for mQTL and MD GWAS shown for each test.

## Data Availability

Summary statistics for MD were obtained from Psychiatric Genomics Consortium (10.6084/m9.figshare.27061255) (ref. ^75^). GoDMC mQTL data were obtained from http://www.godmc.org.uk/. DNAm reference data were obtained from the ‘IlluminaHumanMethylationEPICanno.ilm10b4.hg19’ R package (version 3.13). The annotation object was created on the basis of the product file provided by Illumina for the Infinium MethylationEPIC v1.0 Beadchip (https://support.illumina.com/downloads/infinium-methylationepic-v1-0-product-files.html), with the UCSC gene names provided as the target gene regions of the assay. We searched the EWAS Atlas (https://ngdc.cncb.ac.cn/ewas/atlas) and EWAS Catalog (http://www.ewascatalog.org/) for significantly associated CpGs and genes and the GWAS Catalog (https://www.ebi.ac.uk/gwas/) for annotated genes. According to the terms of consent, access to any form of individual-level data requires application for each individual cohort. Summary statistics of the MWAS meta-analysis are available at 10.7488/ds/7929 and via figshare at 10.6084/m9.figshare.29940299 (ref. ^76^).

## Code availability

The pipeline used for cohort MWAS analysis is publicly available via GitHub (https://github.com/psychiatric-genomics-consortium/mdd-mwas). Scripts used for meta-analysis and downstream analyses can be found at https://github.com/xshen796/MD_metaMWAS_PGCMDDw1.
