# Supplementary material for: A methylome-wide association study of major depression with out-of-sample case–control classification and trans-ancestry comparison
Source: Nat Ment Health. 2025 Sep 16;3(10):1152–67. doi: 10.1038/s44220-025-00486-4 (PMC12504109; doi:10.1038/s44220-025-00486-4)
Supplement: Supplementary file 2 — Reporting Summary [file 44220_2025_486_MOESM2_ESM.pdf]

nature portfolio

Corresponding author(s): *Double-anonymous peer review submissions: write DAPR and your manuscript number here instead of author names.*

Last updated by author(s): *YYYY-MM-DD*

Reporting Summary

Nature Portfolio wishes to improve the reproducibility of the work that we publish. This form provides structure for consistency and transparency in reporting. For further information on Nature Portfolio policies, see our [Editorial Policies] and the [Editorial Policy Checklist].

Statistics

For all statistical analyses, confirm that the following items are present in the figure legend, table legend, main text, or Methods section.

n/a	Confirmed	
☐	☒	The exact sample size (n) for each experimental group/condition, given as a discrete number and unit of measurement
☒	☐	A statement on whether measurements were taken from distinct samples or whether the same sample was measured repeatedly
☐	☒	The statistical test(s) used AND whether they are one- or two-sided<br>*Only common tests should be described solely by name; describe more complex techniques in the Methods section.*
☐	☒	A description of all covariates tested
☐	☒	A description of any assumptions or corrections, such as tests of normality and adjustment for multiple comparisons
☐	☒	A full description of the statistical parameters including central tendency (e.g. means) or other basic estimates (e.g. regression coefficient) AND variation (e.g. standard deviation) or associated estimates of uncertainty (e.g. confidence intervals)
☐	☒	For null hypothesis testing, the test statistic (e.g. F, t, r) with confidence intervals, effect sizes, degrees of freedom and P value noted<br>*Give P values as exact values whenever suitable.*
☒	☐	For Bayesian analysis, information on the choice of priors and Markov chain Monte Carlo settings
☒	☐	For hierarchical and complex designs, identification of the appropriate level for tests and full reporting of outcomes
☐	☒	Estimates of effect sizes (e.g. Cohen's d, Pearson's r), indicating how they were calculated

Our web collection on statistics for biologists contains articles on many of the points above.

Software and code

Policy information about availability of computer code

Data collection

Summary statistics of MWAS for Major Depression were obtained from all 18 cohorts participated in the study. Details for each individual study can be found in Table 1 and Supplementary Information, Cohort details section. Written consents were obtained from all participants. In brief, DNAm data were obtained from DNA extracted from whole blood. Eight studies used the Infinium Human Methylation 450 (450K) BeadChip array (Illumina Inc., number of CpG sites ranged from 275,868 to 438,752 after quality check) and the other ten studies used the Illumina Infinium Methylation EPIC array (Illumina Inc., number of CpG sites ranged from 673,085 to 809,447 after quality check). Life-time diagnosis of MD was derived based on structured clinical interview or self-reported symptoms.

Data analysis

MWAS was conducted in each individual study before the meta-analysis. Linear regression was used to test the association between DNA methylation (M-values, dependent variable) and MD diagnosis (independent variable) using a pipeline available at the URL: https://github.com/psychiatric-genomics-consortium/mdd-mwas. Those cohorts that used their own specific pipelines were specified in the Supplementary Information, Cohort details section.

The pipeline uses the R package 'limma' (version 3.60.6) for linear regression on large omic data. Three models representing increasingly rigorous correction for potential confounders were estimated. Covariates for the simplest model were age, sex, batch, the first 20 methylation principal components (PCs) or surrogate variables (SVs) based on the study protocol for each individual cohort, and white-blood cell proportions estimated from DNA methylation data of CD8+T, CD4+T, natural killer cells, B cells and granulocytes. The AHRR (aryl hydrocarbon receptor repressor)-adjusted model had an additional covariate that adjusted for smoking status by including the M-values for the AHRR probe (cg05575921), due to its known accuracy in predicting smoking and its consistency and availability in all studies. Finally, a third model with additional covariates (referred to as the 'complex model') was fitted that contained body mass index (BMI) and alcohol consumption in addition to all the other covariates included in the previous models. Results for the AHRR model were reported as the main findings.

Meta-analysis of cohort-level MWAS was conducted using METAL (version 2011)59 in a two-stage process. First, meta-analysis was performed on studies that used 450K and EPIC arrays separately, due to the difference of CpG sites available for each array (Figure 1).

For manuscripts utilizing custom algorithms or software that are central to the research but not yet described in published literature, software must be made available to editors and reviewers. We strongly encourage code deposition in a community repository (e.g. GitHub). See the Nature Portfolio guidelines for submitting code & software for further information.

Data

Policy information about availability of data

All manuscripts must include a data availability statement. This statement should provide the following information, where applicable:

- Accession codes, unique identifiers, or web links for publicly available datasets
- A description of any restrictions on data availability
- For clinical datasets or third party data, please ensure that the statement adheres to our policy

Summary statistics for MD was obtained from Psychiatric Genomics Consortium (https://doi.org/10.6084/m9.figshare.27061255). GoDMC mQTL data was obtained from the URL: http://www.godmc.org.uk/. DNAm reference data was obtained from the ‘IlluminaHumanMethylationEPICanno.ilm10b4.hg19’ R package (version 3.13). The annotation object was created based on the product file provided by Illumina for the Infinium MethylationEPIC v1.0 Beadchip (https://support.illumina.com/downloads/infinium-methylationepic-v1-0-product-files.html), with the UCSC gene names provided as the target gene regions of the assay. We searched the EWAS Atlas (https://ngdc.cncb.ac.cn/ewas/atlas) and EWAS Catalog (http://www.ewascatalog.org/) for significantly associated CpGs and genes and the GWAS Catalog (https://www.ebi.ac.uk/gwas/) for annotated genes. According to the terms of consent, access to any form of individual-level data requires application for each individual cohort.
Summary statistics of the MWAS meta-analysis is available at the URL: https://doi.org/10.7488/ds/7929.

Research involving human participants, their data, or biological material

Policy information about studies with human participants or human data. See also policy information about sex, gender (identity/presentation), and sexual orientation and race, ethnicity and racism.

Reporting on sex and gender	We report biological sex throughout in our main text and Supplementary information.
Reporting on race, ethnicity, or other socially relevant groupings	Ethnicity was reported as genetic ancestry in the main text. In summary, we conducted an MWAS meta-analysis for Major Depression using DNA extracted from whole blood in 18 cohorts comprising 24,754 individuals of European ancestry. We also assessed whether effect sizes for significant findings in European samples were positively associated with thos in an independent East Asian sample.
Population characteristics	A total of 24,754 European-ancestry participants (5,443 cases) from 18 studies were included in the meta-analysis. The mean age of participants in each study ranged from 15 to 59 years. Details for each individual study can be found in Table 1 and Supplementary information, Cohort details.
Recruitment	Twelve of the cohorts included in our study are population cohorts and self-selection bias may present in these cohorts.
Ethics oversight	The study was approved by the NHS Tayside Research Ethics committee (05/s1401/89). Ethics declaration for each participating cohort can be found in the Supplementary information.

Note that full information on the approval of the study protocol must also be provided in the manuscript.

Field-specific reporting

Please select the one below that is the best fit for your research. If you are not sure, read the appropriate sections before making your selection.

☒ Life sciences ☐ Behavioural & social sciences ☐ Ecological, evolutionary & environmental sciences

For a reference copy of the document with all sections, see nature.com/documents/nr-reporting-summary-flat.pdf

Life sciences study design

All studies must disclose on these points even when the disclosure is negative.

Sample size	Sample sizes were not predetermined before the study due to a lack of findings in previous studies leading to insufficient information to determine sample sizes. However, the present study comprises 18 studies and a total of 24,754 individuals, which is the largest MWAS for Major Depression to date and over twice the size of any previous MWAS for depression or depressive symptoms.
Data exclusions	MWAS meta-analysis was conducted on summary statistics shared by each individual study. Data exclusion criteria were described in detail in Supplementary information, Cohort details. In brief, similar quality check procedures were used for all studies, including removing probes with outlying signal intensity, bead count and detection p-values, removing participants with mismatched sex prediction from DNA methylation data and removing cross-hybridising probes that map to common genetic variants (at MAF > 0.05) and polymorphic probes.<br>At the meta-analysis stage, those CpG sites that were either available for more than half of the studies using the given array or had a total sample size over 80% of the max sample size were kept for further analysis. CpG sites with excessive standard errors (SE>0.5, see Supplementary Figure 1) were removed from analysis.

Replication	Replication of meta-MWAS was conducted via leave-one-out meta-analysis and out-of-sample methylation score classification. Replication for Mendelian Randomisation was conducted by using two independent methQTL datasets (GoDMC for discovery analysis and Generation Scotland for replication). Causal effects of DNAm to Major Depression were tested using these two independent datasets. Out of the 23 potentially causal effects found in the discovery analysis, four CpG sites were specific to the EPIC array, while 19 were available on both array types and could, therefore, be included in the replication analysis.
Randomization	Participants were allocated to case and control groups based on their diagnosis for Major Depression (MD). Life-time diagnosis of MD was derived based on structured clinical interview or self-reported symptoms. Those studies that derived diagnoses of MD based on structural clinical interviews used criteria from The Diagnostic and Statistical Manual of Mental Disorders, Fifth/Fourth Edition (DSM-5/DSM-4)55. Self-declared MD was based on depressive symptoms lasting for more than two weeks and help-seeking due to depression. Studies that derived MD diagnosis based on multiple time points defined cases as those who experienced any depressive episodes during their lifetime, and controls were those who did not declare MD throughout. A total of 9 studies defined MD cases using structural clinical interview (N cases = 3,790), 4 studies used DSM-criteria questionnaires (N cases = 586), 3 studies used self-administered questionnaires for depressive symptoms (N cases = 528), and 3 studies defined MD cases based on self-declared visits to general practitioner (N cases = 539). Details for MD diagnosis for each cohort can be found in Table 1 and the Supplementary Information, Cohort details.
Blinding	Individual-level DNAm and diagnoses for Major Depression were anonymized, and association analysis for each study was conducted using a pipeline that takes individual-level DNAm and diagnosis as inputs to minimize potential bias. Individual study sites then shared summary statistics for inclusion in the meta-MWAS. The meta-analysis was conducted in a double-blind manner as it was independent from the association analyses within individual studies.

Reporting for specific materials, systems and methods

We require information from authors about some types of materials, experimental systems and methods used in many studies. Here, indicate whether each material, system or method listed is relevant to your study. If you are not sure if a list item applies to your research, read the appropriate section before selecting a response.

Materials & experimental systems

n/a	Involved in the study
☒	☐ Antibodies
☒	☐ Eukaryotic cell lines
☒	☐ Palaeontology and archaeology
☒	☐ Animals and other organisms
☒	☐ Clinical data
☒	☐ Dual use research of concern
☐	☐ Plants

Methods

n/a	Involved in the study
☒	☐ ChIP-seq
☒	☐ Flow cytometry
☒	☐ MRI-based neuroimaging

Plants

Seed stocks	Not applicable
Novel plant genotypes	Not applicable
Authentication	Not applicable
